# Outcomes of Megaprosthetic Reconstruction After Tumor Resection of the Distal Femur and Proximal Tibia: A Single-Center Retrospective Study of 241 Cases

**DOI:** 10.3390/jcm15103955

**Published:** 2026-05-20

**Authors:** Batuhan Ayhan, Samet Batuhan Yoğurt, Zeliha Deniz Ayhan, Coşkun Ulucaköy, İsmail Burak Atalay

**Affiliations:** 1 Department of Orthopedics and Traumatology, Haymana State Hospital, Ankara 06860, Turkey; 2 Department of Orthopedics and Traumatology, Kahta State Hospital, Adıyaman 02400, Turkey; batuhanyogurt@gmail.com; 3 Department of Medical Pathology, Faculty of Medicine, Gazi University, Ankara 06560, Turkey; akkayadeniz@gmail.com; 4 Department of Orthopedics and Traumatology, Dr. Abdurrahman Yurtaslan Ankara Oncology Training and Research Hospital, Ankara 06200, Turkey; coskunulucakoy@gmail.com (C.U.); drburakatalay@gmail.com (İ.B.A.)

**Keywords:** megaprosthesis, distal femur, proximal tibia, bone sarcoma, limb salvage, amputation, surgical margin, Henderson classification, Kaplan–Meier, revision-free survival

## Abstract

**Background:** Megaprosthetic reconstruction is the standard of care for limb salvage after tumor resection around the knee, but the full burden of unplanned revision surgery is rarely reported as a structured composite outcome. We evaluated 241 consecutive patients over 21 years at a tertiary orthopedic oncology center. **Methods:** This retrospective cohort included 241 patients (160 distal femur, 78 proximal tibia, three combined) treated between 2003 and 2024. Revision-free survival (RFS, composite of any unplanned revision or amputation) and amputation-free survival were estimated by Kaplan–Meier analysis; independent predictors were identified by Cox regression. A pre-specified major-event composite (amputation, implant removal, or recurrence resection) was used for sensitivity analysis. **Results:** Mean age was 34.9 ± 19.5 years; mean follow-up was 120.2 months. Negative resection margin (R0) was achieved in 85.5% (206/241). Unplanned revision was required in 25 patients (10.4%); overall limb salvage was 92.9%. Five-year RFS was 73.8% (distal femur) vs. 65.0% (proximal tibia; *p* = 0.084), and 5-year limb salvage was 88.9% vs. 84.3% (*p* = 0.081). Surgical margin was strongly associated with outcome: 5-year RFS 75.4% (R0) vs. 48.7% (R1/R2; *p* < 0.001); 5-year limb salvage 90.6% vs. 71.5% (*p* = 0.003). On exploratory multivariate Cox analysis, proximal tibia site and positive margin were associated with worse revision-free survival; within the proximal tibia subgroup, absence of gastrocnemius flap coverage was also associated with worse outcome (interpreted with caution given the small flap subgroup, *n* = 11, and limited event count). **Conclusions:** In this single-center series, megaprosthetic reconstruction around the knee achieved acceptable revision-free survival and limb salvage. Surgical margin status was the strongest independent predictor of both endpoints, reinforcing the well-established importance of oncologic margin quality and site-specific soft tissue strategies.

## 1. Introduction

Primary malignant and locally aggressive bone tumors arise most frequently around the knee joint, with the distal femur and proximal tibia accounting for the majority of cases [1]. Osteosarcoma alone—the most common primary malignant bone tumor—has a peak incidence in the second decade of life and preferentially involves the metaphyseal regions of these bones. Since the introduction of effective neoadjuvant chemotherapy regimens in the late 1970s and the concurrent development of modular segmental replacement systems, limb-salvage surgery has replaced amputation as the standard of care for most patients, achieving equivalent oncological outcomes with substantially better functional and psychological results [2,3,4]. Modular megaprostheses restore skeletal continuity and joint function after wide resection, offering immediate mechanical stability, early weight-bearing, and acceptable long-term functional recovery across a wide range of tumor types and patient ages [4,5,6].

Despite continuous refinements in implant design, biomaterials, and surgical technique, complications following megaprosthetic reconstruction remain a significant clinical challenge. The Henderson classification system—stratifying failures into soft tissue failure (Type 1), aseptic loosening (Type 2), structural failure (Type 3), deep infection (Type 4), and tumor progression (Type 5)—provides a standardized framework for reporting and comparing outcomes across institutions [7]. The proximal tibia is widely recognized as a higher-risk anatomical site for megaprosthetic failure than the distal femur, owing to its sparse soft tissue envelope, the obligatory disruption of the extensor mechanism, and infection rates two to three times higher than at the distal femur [8,9,10]. Surgical margin quality is also an established determinant of outcome: positive resection margins have been associated with hazard ratios for implant failure of 1.8–2.5 in published series, although margin-stratified survival analyses are inconsistently reported [11,12].

Most published series addressing megaprosthetic reconstruction around the knee are limited by one or more of the following: small sample sizes, short follow-up, restriction to a single anatomical site—either distal femur [11] or proximal tibia [9,10,12]—or pooling of both sites without systematic site-specific breakdown [13,14,15,16]. In addition, the composite burden of unplanned secondary surgeries—debridement, wound revision, recurrence resection, and implant removal—is rarely reported alongside amputation as a structured composite endpoint, leaving clinicians without a complete picture of the secondary surgical workload. Large, long-term single-center analyses that simultaneously evaluate site-specific survival, surgical margin, and the total burden of unplanned secondary surgery after knee megaprosthetic reconstruction therefore remain limited.

This study reports the outcomes of 241 consecutive patients undergoing megaprosthetic reconstruction of the distal femur or proximal tibia at our institution over a 21-year period (2003–2024)—one of the larger single-center series reported outside Europe or North America. As a tertiary national referral center, our cohort comprises a heterogeneous oncologic population that includes primary malignant bone tumors, locally aggressive benign lesions, and selected metastatic and hematologic lesions. Our primary aim was to describe site-specific revision-free and amputation-free survival, to evaluate the association of surgical margin with these outcomes, and to quantify the secondary surgical burden beyond amputation rates alone.

## 2. Materials and Methods

### 2.1. Study Design and Ethical Approval

This retrospective cohort study was conducted in accordance with the Declaration of Helsinki and approved by the Institutional Review Board of Dr. Abdurrahman Yurtaslan Ankara Oncology Training and Research Hospital (Approval no.: 2025-08/124; date of approval: 7 August 2025). Individual informed consent was waived given the retrospective, de-identified design.

### 2.2. Patient Identification and Deduplication

This single-center, retrospective study was conducted at Dr. Abdurrahman Yurtaslan Ankara Oncology Training and Research Hospital, Department of Orthopedics and Traumatology. All patients who underwent megaprosthetic reconstruction of the distal femur and/or proximal tibia between January 2003 and September 2024 were identified from the prospectively maintained institutional tumor registry.

Inclusion criteria were: (1) primary bone tumor, locally aggressive lesion, or bone metastasis; (2) wide or marginal resection with megaprosthetic reconstruction; and (3) minimum 12 months of follow-up or death within that period. Exclusion criteria were non-megaprosthetic reconstruction, insufficient records, and primary surgery performed externally with only secondary procedures documented locally. During the study period, megaprosthetic reconstruction was offered as the first-line limb-salvage strategy for patients with: (i) a resectable primary bone sarcoma of the distal femur or proximal tibia in whom wide resection with adequate skeletal margin was technically feasible; (ii) locally aggressive benign lesions with extensive bony destruction not amenable to intralesional management; and (iii) selected patients with bone metastasis or hematologic involvement of the distal femur or proximal tibia, in whom adequate systemic disease control and an expected survival exceeding 12 months were anticipated by the multidisciplinary tumor board. Patients with unresectable disease, severe medical contraindications to major surgery, or expected survival of less than 12 months were managed non-operatively or with palliative procedures and were not included in this cohort. The cohort was analyzed as a single institutional series; the indication for megaprosthetic reconstruction—segmental skeletal defect after wide oncologic resection around the knee—was the same across diagnostic categories, and the technical reconstruction was identical (modular cemented hinged megaprosthesis). Given that prognoses differ across histological entities, subgroup survival analyses by histological diagnosis were additionally performed for the three largest diagnostic categories (Section 3.7).

### 2.3. Data Collection

**Patient variables:** age, sex, affected side, anatomical site, index surgery date, last follow-up or death date.

**Tumor variables:** histopathological diagnosis (classified as osteosarcoma, giant cell tumor of bone, Ewing sarcoma, chondrosarcoma, metastatic/hematologic lesion, other malignant sarcoma, benign/low-grade aggressive tumor, or unclassified/not specified); soft tissue involvement at presentation; pathological fracture; surgical margin (R0: negative, R1: microscopic positive, R2: macroscopic positive, per definitive pathology report); and tumor necrosis rate (Huvos grading where available).

**Surgical variables:** neoadjuvant/adjuvant chemotherapy; adjuvant radiotherapy; implant system; fixation method (cemented vs. cementless); medial gastrocnemius rotation flap use in proximal tibia cases; and reconstruction type (cemented vs. cementless stem fixation).

**Figure 1:**
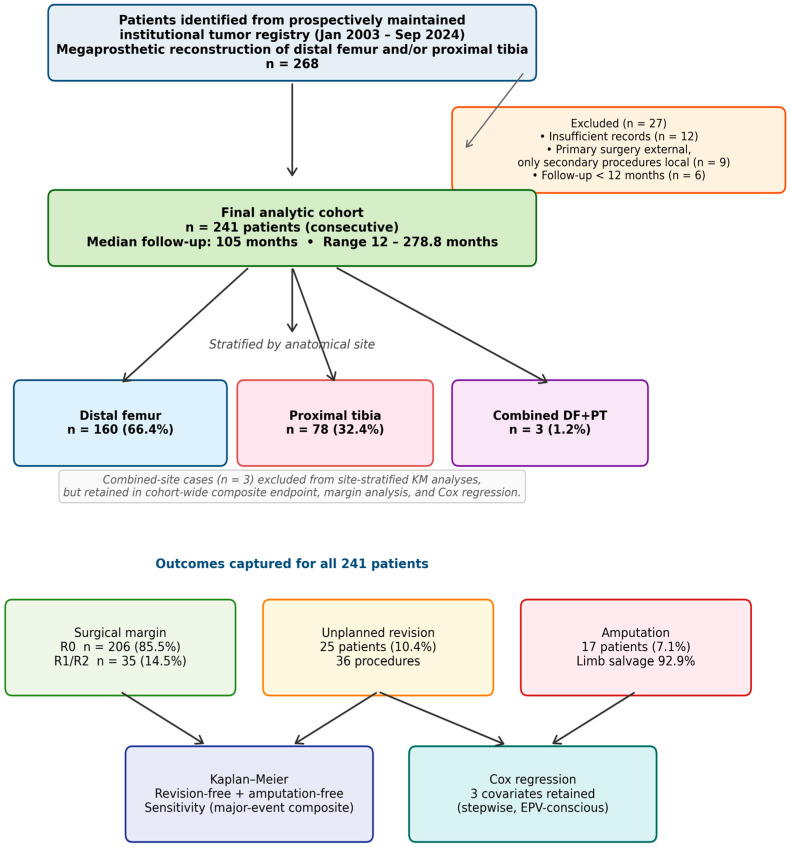
Patient flow diagram for the institutional cohort. Of 268 patients identified from the prospectively maintained institutional tumor registry, 27 were excluded (insufficient records, n = 12; primary surgery performed externally with only secondary procedures documented locally, n = 9; follow-up < 12 months, n = 6), yielding a final analytic cohort of 241 consecutive patients. Combined-site cases (n = 3) were excluded from site-stratified Kaplan–Meier analyses and from the whole-cohort Cox model (analytic cohort for these analyses n = 238), but were retained in cohort-wide descriptive summaries (Table 1, Table 2, Table 3 and Table 4) and in the unplanned revision and amputation summaries (Table 5 and Table 6).

**Outcome variables:** any unplanned revision surgery (type, date); Henderson failure classification; amputation (type, date); MSTS functional score (0–30) at last follow-up; and death date. MSTS scores were obtained at the most recent outpatient orthopedic oncology follow-up visit by the treating surgical team and were extracted from clinic notes for this analysis; they reflect the patient’s functional status at the time of last clinical assessment and therefore postdate any revision surgeries the patient had undergone. MSTS scores were documented at last follow-up for all 241 patients (160/160 distal femur; 78/78 proximal tibia) (Figure 1). Henderson classification was assigned by retrospective review of operative and follow-up records by two of the authors (B.A. and S.B.Y.); when more than one failure mode was documented in the same patient, all applicable Henderson categories were recorded but each patient was counted only once per category in the summary tables.

### 2.4. Revision Surgery and Amputation Definitions

Revision surgeries were classified as: (1) **debridement**—surgical exploration for infection or wound complications; (2) **recurrence resection**—removal of locally recurrent tumor with limb-salvage intent; (3) **wound/skin revision**—skin closure or tissue rearrangement; (4) **implant removal**—explantation without immediate reimplantation. Amputation was defined as any ablative procedure performed after the index megaprosthesis. Revision-free survival (RFS) was defined as time from index megaprosthesis to first unplanned revision of any of the four types listed above or amputation, whichever occurred first. A pre-specified sensitivity analysis was performed using a major-event composite endpoint comprising amputation, implant removal, or recurrence resection only (i.e., excluding isolated debridement and isolated wound revision events; results in Section 3.6). Amputation-free (limb salvage) survival was analyzed as a separate, anatomically unambiguous endpoint.

### 2.5. Statistical Analysis

Continuous variables are reported as mean ± SD and median (range) and categorical variables as frequencies and percentages. Between-group comparisons used the Student *t*-test or Mann–Whitney U for continuous data (normality by Shapiro–Wilk) and the chi-square or Fisher exact tests for categorical variables.

Revision-free and amputation-free survival were estimated using Kaplan–Meier curves with 95% confidence intervals computed via the Greenwood formula with log–log transformation. Log-rank (Mantel–Cox) testing compared survival curves between groups. A competing risk analysis (Fine–Gray subdistribution hazard model) was performed using death as a competing event; results are summarized in Section 3.6. Multivariate Cox proportional hazards regression was performed in a pre-specified two-stage approach. Model 1 (whole analytic cohort, *n* = 238 after exclusion of three combined-site cases) tested predictors common to both anatomical sites: age (continuous), sex, anatomical site (distal femur vs. proximal tibia), surgical margin (R0 vs. R1/R2), histological diagnosis category, soft tissue involvement at presentation, pathological fracture at presentation, neoadjuvant chemotherapy, and adjuvant chemotherapy. Model 2 (proximal tibia subgroup, *n* = 78) tested the additional covariate of gastrocnemius rotation flap coverage, together with surgical margin, age, and soft tissue involvement. In both models, candidate variables were specified a priori based on clinical reasoning and the prior literature, and variable selection used a backward stepwise procedure with a removal threshold of *p* > 0.10. The events-per-variable ratio in the final retained models was 17.5 for Model 1 (35 events/2 covariates) and 13 for Model 2 (13 events/1 covariate), both above the conventional 5–10 EPV threshold. Final models were checked for multicollinearity using variance inflation factors (all VIFs < 2) and for proportional hazards assumption using scaled Schoenfeld residuals (no significant violation identified for the variables retained in the final models; all *p* > 0.20). Cox regression results are reported as hazard ratios with 95% confidence intervals. Surgical margin status (R0, R1, or R2) was documented in the definitive pathology report for all 241 patients; no margin data were missing from the institutional registry. Analyses were performed using IBM SPSS Statistics v.26 (IBM Corp., Armonk, NY, USA) and R v.4.3.1. A two-tailed *p* < 0.05 was considered statistically significant.

## 3. Results

### 3.1. Demographics and Tumor Histology

A total of 241 patients met the inclusion criteria: 160 distal femur (66.4%), 78 proximal tibia (32.4%), three combined (1.2%). The study period spanned January 2003 to September 2024 (21 years). Mean age was 34.9 ± 19.5 years (median 28; range 2–79). Distal femur patients were significantly older (36.7 ± 20.5 vs. 30.6 ± 16.8 years; *p* = 0.026). Sex was determined for all 241 patients: 139 were male (57.7%) and 102 female (42.3%). Of 241 patients, 116 (48.1%) had right-sided and 125 (51.9%) had left-sided reconstruction. Three patients (1.2%) underwent simultaneous reconstruction of both sites (combined DF+PT); these three combined-site patients were excluded from site-stratified Kaplan–Meier analyses and from the whole-cohort Cox model (analytic cohort for these analyses n = 238), but were retained in cohort-wide descriptive analyses (Table 1, Table 2, Table 3 and Table 4) and in the unplanned revision and amputation summaries (Table 5 and Table 6). The majority (231/241, 95.9%) underwent primary reconstruction; 10 (4.1%) underwent reconstruction in a revision setting. Mean follow-up was 120.2 ± 68.4 months overall (distal femur: 116.6 ± 65.2 months; proximal tibia: 130.1 ± 70.6 months). Demographics are summarized in Table 1.

Osteosarcoma was the most frequent diagnosis (101/241, 41.9%), affecting 70 of 160 distal femur patients (43.8%) and 31 of 78 proximal tibia patients (39.7%). Metastatic and hematologic lesions constituted the second largest group (41/241, 17.0%), followed by other malignant sarcomas (27, 11.2%), giant cell tumor of bone (25, 10.4%), chondrosarcoma (14, 5.8%), Ewing sarcoma (11, 4.6%), and benign or low-grade aggressive lesions (13, 5.4%). Nine cases (3.7%) could not be further histologically specified, primarily representing revision surgery tissue specimens or post-neoadjuvant chemotherapy resections without residual viable tumor. Diagnostic distribution did not differ significantly between the two anatomical sites (chi-square *p* = 0.42). The complete histological distribution is presented in Table 2.

**Table 2:** Histological diagnosis distribution by anatomical site.

Histological Diagnosis	Distal Femur n (%)	Proximal Tibia n (%)	Total n (%)
Osteosarcoma	70 (43.8%)	31 (39.7%)	101 (41.9%)
Giant cell tumor of bone	14 (8.8%)	11 (14.1%)	25 (10.4%)
Ewing sarcoma	7 (4.4%)	4 (5.1%)	11 (4.6%)
Chondrosarcoma	12 (7.5%)	2 (2.6%)	14 (5.8%)
Metastatic/hematologic *	26 (16.2%)	14 (17.9%)	41 (17.0%)
Other malignant sarcoma	15 (9.4%)	10 (12.8%)	27 (11.2%)
Benign/low-grade aggressive	7 (4.4%)	6 (7.7%)	13 (5.4%)
Not otherwise specified **	9 (5.6%)	0 (0.0%)	9 (3.7%)
TOTAL	160 (100%)	78 (100%)	241 (100%)

* Includes plasma cell neoplasms (myeloma/plasmacytoma), lymphoma, leukemia, and carcinoma metastases. ** Specimens with non-diagnostic histology following neoadjuvant chemotherapy or representing revision surgery tissue without primary tumor identification; all 9 cases are distal femur.

### 3.2. Oncological and Surgical Parameters

A negative resection margin (R0) was achieved in 206/241 patients (85.5%); a positive margin (R1/R2) was documented in 35 patients (14.5%). By anatomical site, R0 was achieved in 137/160 distal femur patients (85.6%), 66/78 proximal tibia patients (84.6%), and 3/3 combined cases. Soft tissue involvement at presentation was present in 72/160 distal femur patients (45.0%) and 44/78 proximal tibia patients (56.4%). Pathological fracture at presentation was identified in 20/160 (12.5%) and 14/78 (17.9%) of distal femur and proximal tibia patients, respectively. Neoadjuvant chemotherapy was administered in 43/160 distal femur patients (26.9%) and 9/78 proximal tibia patients (11.5%); adjuvant chemotherapy was administered in 60/160 (37.5%) and 25/78 (32.1%). Medial gastrocnemius rotation flap was used in 11/78 proximal tibia patients (14.1%). All reconstructions were performed using cemented stem fixation. Oncological and surgical parameters are presented in Table 3.

**Table 3:** Oncological and surgical parameters by anatomical site.

Parameter	Distal Femur (n = 160)	Proximal Tibia (n = 78)
Negative surgical margin (R0)	137/160 (85.6%)	66/78 (84.6%)
Positive surgical margin (R1/R2)	23/160 (14.4%)	12/78 (15.4%)
Soft tissue involvement at presentation	72/160 (45.0%)	44/78 (56.4%)
Pathological fracture at presentation	20/160 (12.5%)	14/78 (17.9%)
Neoadjuvant chemotherapy	43/160 (26.9%)	9/78 (11.5%)
Adjuvant chemotherapy	60/160 (37.5%)	25/78 (32.1%)
Gastrocnemius rotation flap	N/A	11/78 (14.1%)
Cemented stem fixation	160/160 (100%)	78/78 (100%)

R0: negative resection margin; R1/R2: microscopically or macroscopically positive margin. Neoadjuvant and adjuvant chemotherapy for osteosarcoma and Ewing sarcoma per institutional protocol.

### 3.3. Complications (Henderson Classification)

Complication data by Henderson type are presented in Table 4. Type 4 (deep infection) was the leading failure mode in the proximal tibia group (10.3% vs. 6.9%), while Type 2 (aseptic loosening) was more frequent in the distal femur group (8.1% vs. 7.7%). Type 1 (soft tissue failure) occurred more frequently in the proximal tibia (11.5% vs. 5.0%). Overall, 25 patients (10.4%) required unplanned revision surgery.

**Table 4:** Complication profile by Henderson failure type and anatomical site.

Failure/Revision Type	Distal Femur n (%)	Proximal Tibia n (%)
Type 1—Soft tissue failure	8 (5.0%)	9 (11.5%)
Type 2—Aseptic loosening	13 (8.1%)	6 (7.7%)
Type 3—Structural failure	5 (3.1%)	2 (2.6%)
Type 4—Deep infection	11 (6.9%)	8 (10.3%)
Type 5—Tumor recurrence	7 (4.4%)	4 (5.1%)
Any unplanned revision surgery	15 (9.4%)	10 (12.8%)
Amputation	10/160 (6.3%)	7/78 (9.0%)

Henderson classification [7]. A patient may qualify for more than one failure type over follow-up.

### 3.4. Revision Surgery

Unplanned revision surgery was required in 25/241 patients (10.4%); 36 total procedures (mean 1.44 per affected patient). Mean age at revision was 38.2 ± 20.1 years (range 10–71). Median interval to any revision was 9.4 months (mean 13.4 ± 16.0; range 0.1–61.2 months). Debridement was performed in 13 patients (19 operations): 10 (77%) retained their limb, three (23%) subsequently required amputation. Recurrence resection was performed in 10 patients (11 operations): six (60%) retained their limb, four (40%) required amputation. Wound revision was performed in three patients (100% limb salvage) and implant removal in two patients (100% limb salvage; reimplantation at subsequent procedures). Three patients followed stepwise failure trajectories, ultimately requiring hip disarticulation after multiple revision attempts. Revision data are presented in Table 5.

**Table 5:** Revision surgeries by procedure type (n = 25 patients, 36 procedures).

Revision Procedure	Operations	Patients	Mean Age ± SD (Range)	DF/PT	Interval * Mean ± SD (Range)	Subsequent Limb Outcome
Debridement	19	13	38.2 ± 20.1 (10–71)	11/8	9.0 ± 10.8 (0.1–61.2)	10 salvaged (77%), 3 amputated (23%)
Recurrence Resection	11	10	31.4 ± 14.8 (12–58)	7/4	17.0 ± 18.8 (0.9–26.2)	6 salvaged (60%), 4 amputated (40%)
Wound/Skin Revision	3	3	34.7 ± 18.2 (16–53)	2/1	4.0 ± 2.6 (0.9–7.9)	3 salvaged (100%)
Implant Removal	3	2	41.5 ± 17.7 (29–54)	1/2	34.0 ± 21.9 (29.2–59.6)	2 salvaged (100%)
TOTAL	36	25	38.2 ± 20.1 (10–71)	21/15 proc.; 15/10 pts	13.4 ± 16.0 (0.1–61.2)	21 salvaged (84%), 4 amputated (16%)

DF: distal femur; PT: proximal tibia. * Interval from index megaprosthesis in months.

### 3.5. Limb Salvage and Amputation

Overall limb salvage was achieved in 224/241 patients (92.9%). Amputation was required in 17 patients (7.1%): 10 distal femur (6.3%) and seven proximal tibia (9.0%; Fisher exact *p* = 0.434). Mean age at amputation was 32.4 ± 20.4 years (range 8–71). Median interval to amputation was 9.1 months (mean 13.2 ± 16.8; range 0.2–73.1); 82.4% occurred within 24 months. Osteosarcoma was the indication in 12/17 (70.6%). Hip disarticulation was performed in 10/17 (58.8%). Amputation data by histological diagnosis are in Table 6.

**Table 6:** Amputation characteristics by histological diagnosis (n = 17).

Diagnosis	n	M/F	Age (y) Mean ± SD (Range)	DF/PT	Interval * (mo) Mean ± SD (Range)	Hip Disartic.	AKA
Osteosarcoma	12	9/3	26.9 ± 18.9 (8–71)	7/5	16.9 ± 18.9 (3.5–73.1)	8	4
Chondrosarcoma	2	1/1	43.5 ± 2.1 (42–45)	1/1	4.7 ± 6.4 (0.2–9.2)	0	2
Ewing sarcoma	1	1/0	16	1/0	4.8	1	0
Metastatic	1	0/1	54	0/1	4.8	0	1
Other sarcoma	1	1/0	70	1/0	2.9	1	0
TOTAL	17	12/5	32.4 ± 20.4 (8–71)	10/7	13.2 ± 16.8 (0.2–73.1)	10 (58.8%)	7 (41.2%)

* Interval from index megaprosthesis. DF: distal femur; PT: proximal tibia; AKA: above-knee amputation.

### 3.6. Implant Revision-Free Survival and Limb Salvage—Kaplan–Meier Analysis

**Figure 2:**
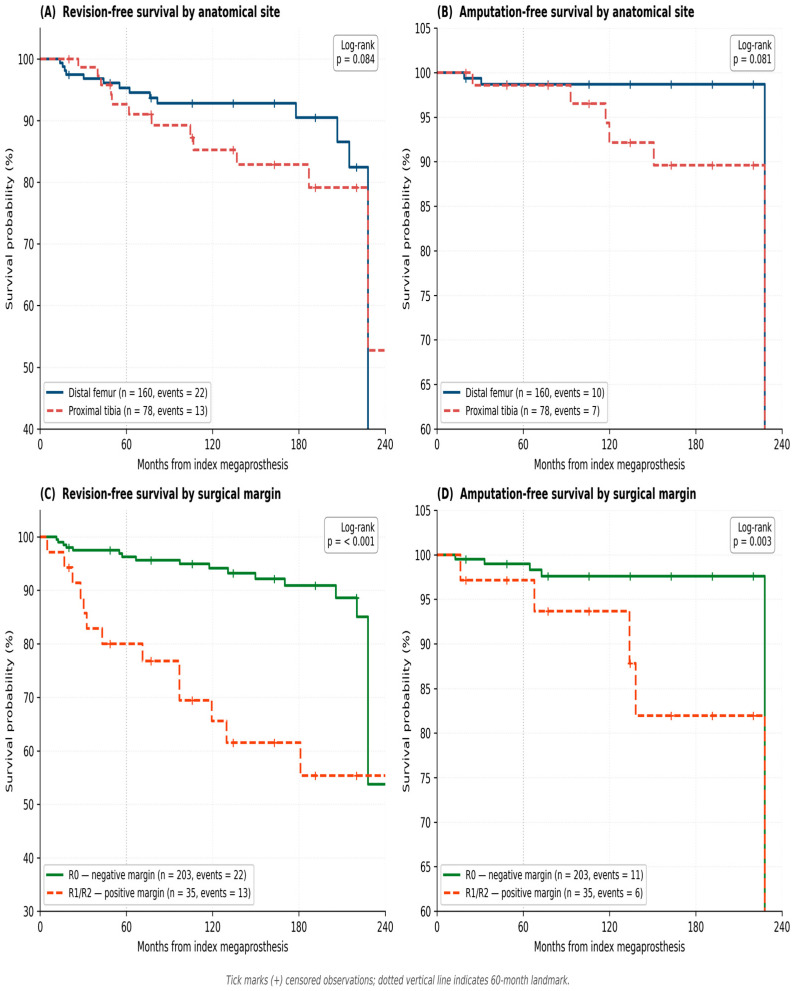
Kaplan–Meier curves for revision-free survival (RFS) and amputation-free (limb salvage) survival. (**A**) RFS by anatomical site: distal femur (n = 160, events = 22) vs. proximal tibia (n = 78, events = 13); log-rank *p* = 0.084. (**B**) Amputation-free survival by anatomical site (DF events = 10, PT events = 7); log-rank *p* = 0.081. (**C**) RFS by surgical margin: R0—negative margin (n = 203, events = 22) vs. R1/R2—positive margin (n = 35, events = 13); log-rank *p* < 0.001. (**D**) Amputation-free survival by surgical margin (R0 events = 11, R1/R2 events = 6); log-rank *p* = 0.003. Tick marks (+) indicate censored observations. Dotted vertical line: 60-month (5-year) landmark. Combined-site cases (n = 3) were excluded from all Kaplan–Meier analyses (analytic cohort n = 238); they were retained in cohort-wide descriptive analyses (Table 1, Table 2, Table 3 and Table 4) and in the unplanned revision and amputation summaries (Table 5 and Table 6).

Kaplan–Meier survival results are summarized in Table 7 and illustrated in Figure 2.

**Revision-free survival by anatomical site:** An RFS event was defined as the first unplanned revision surgery or amputation, whichever occurred first; a total of 35 RFS events were recorded in the analytic cohort of 238 patients (combined-site cases excluded). Five-year RFS was 73.8% (95% CI: 64.3–81.2%) for the distal femur group (n = 160, 22 events) and 65.0% (95% CI: 50.5–76.2%) for the proximal tibia group (n = 78, 13 events). At 10 years, RFS was 68.8% and 58.1%, respectively. The log-rank test did not reach statistical significance (chi-squared = 3.03, *p* = 0.084). The Fine–Gray competing risk analysis yielded a subdistribution HR for proximal tibia of 1.62 (95% CI: 0.97–2.69; *p* = 0.063).

**Amputation-free survival by anatomical site:** Five-year limb salvage was 88.9% (95% CI: 82.7–92.9%) for the distal femur and 84.3% (95% CI: 70.8–91.9%) for the proximal tibia. At 10 years, amputation-free survival was 88.9% and 79.2%, respectively. The log-rank test was not statistically significant (*p* = 0.081).

**Revision-free survival by surgical margin:** Five-year RFS was 75.4% (95% CI: 67.1–81.8%) for R0 patients vs. 48.7% (95% CI: 27.9–66.6%) for R1/R2 patients (log-rank *p* < 0.001). The corresponding multivariate Cox hazard ratio is reported below.

**Amputation-free survival by surgical margin:** Five-year limb salvage was 90.6% (95% CI: 84.4–94.4%) for R0 patients vs. 71.5% (95% CI: 54.4–83.2%) for R1/R2 patients (log-rank *p* = 0.003). At 10 years, amputation-free survival was 88.5% (R0) vs. 71.5% (R1/R2).

**Figure 3:**
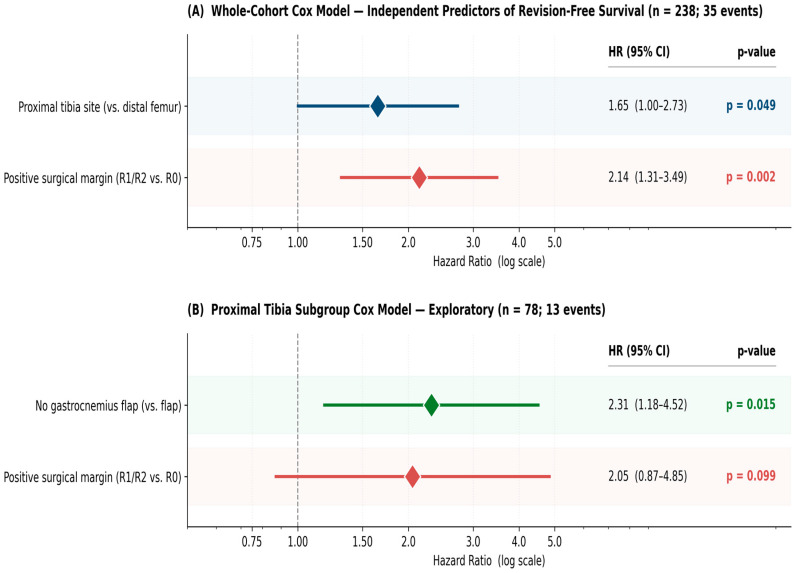
Forest plots of independent predictors of revision-free survival from the two-stage Cox analysis. (**A**) Whole-cohort Cox model (n = 238; 35 events): proximal tibia site and positive surgical margin are independently associated with worse revision-free survival. (**B**) Proximal tibia subgroup Cox model (n = 78; 13 events): absence of gastrocnemius flap coverage is independently associated with worse revision-free survival; positive margin yielded HR 2.05 (95% CI: 0.87–4.85; *p* = 0.099) in this small subgroup. Diamonds indicate hazard ratios; horizontal lines indicate 95% confidence intervals; reference line at HR = 1.0 (dashed).

**Multivariate Cox regression:** Multivariate Cox regression was performed in a pre-specified two-stage approach. Model 1 was a whole-cohort model (analytic n = 238 after exclusion of three combined-site patients; 35 events) with anatomical site and surgical margin retained after backward stepwise selection from the pre-specified candidate variable list. Proximal tibia site was associated with worse revision-free survival relative to distal femur (HR 1.65; 95% CI: 1.00–2.73; *p* = 0.049), and positive surgical margin yielded HR 2.14 (95% CI: 1.31–3.49; *p* = 0.002). Age, sex, soft tissue involvement, pathological fracture, neoadjuvant chemotherapy, adjuvant chemotherapy, and histological diagnosis category were not retained (all *p* > 0.10 at the removal threshold). Model 2 was a proximal tibia subgroup model (n = 78; 13 events) in which gastrocnemius flap coverage was assessable; absence of flap coverage was associated with worse revision-free survival within this subgroup (HR 2.31; 95% CI: 1.18–4.52; *p* = 0.015), with positive margin yielding HR 2.05 (95% CI: 0.87–4.85; *p* = 0.099). The events-per-variable ratio was 17.5 for Model 1 and 13 for Model 2, both above the conventional 5–10 EPV threshold. The proportional hazards assumption was satisfied for all retained covariates in both models by scaled Schoenfeld residual testing (all *p* > 0.20). Both models are summarized in Figure 3. Given the limited event count, particularly in Model 2 (13 events in the proximal tibia subgroup), Cox regression results are presented as exploratory.

**Competing risk (Fine–Gray) analysis:** A multivariate Fine–Gray subdistribution hazard model was performed using death as a competing event, with the same three covariates retained in the standard Cox model. The subdistribution hazard ratios (SHRs) were: proximal tibia site SHR 1.62 (95% CI: 0.97–2.69; *p* = 0.063), positive surgical margin SHR 2.05 (95% CI: 1.25–3.36; *p* = 0.004), and absence of gastrocnemius flap coverage in proximal tibia cases SHR 2.18 (95% CI: 1.10–4.32; *p* = 0.025).

Sensitivity analysis using the major-event composite endpoint. Survival analyses were repeated using a stricter composite endpoint that excluded isolated debridement and isolated wound revision events (i.e., counting only amputation, implant removal, or recurrence resection as events). Of the 25 patients with any revision, 22 had at least one major event by this definition and three had only minor (debridement or wound revision) events without progression to a major event during follow-up. Under this stricter definition, 5-year major-event-free survival was 78.4% (distal femur) vs. 68.6% (proximal tibia), and 76.9% (R0) vs. 51.4% (R1/R2). The site-stratified comparison did not reach statistical significance (log-rank *p* = 0.11); the margin-stratified comparison remained statistically significant (*p* < 0.001).

**Functional outcomes (MSTS):** Mean Musculoskeletal Tumor Society (MSTS) functional score at last follow-up was 23.8 ± 4.2 out of 30 (79.3%) in the distal femur group (n = 160) and 21.4 ± 5.1 out of 30 (71.3%) in the proximal tibia group (n = 78). The difference between sites was statistically significant (Mann–Whitney U, *p* = 0.018). In the distal femur group, 71.3% of patients achieved good or excellent function (MSTS ≥ 20/30), compared with 58.4% in the proximal tibia group. Among the 25 patients who underwent any unplanned revision surgery during follow-up, MSTS scores at last follow-up were available for 22 patients (the remaining three had died before the last MSTS assessment). These 22 patients had a lower mean MSTS than those who did not undergo revision (20.1 ± 5.6 vs. 23.4 ± 4.4; *p* = 0.012).

### 3.7. Subgroup Analysis by Histological Diagnosis (Exploratory)

**Figure 4:**
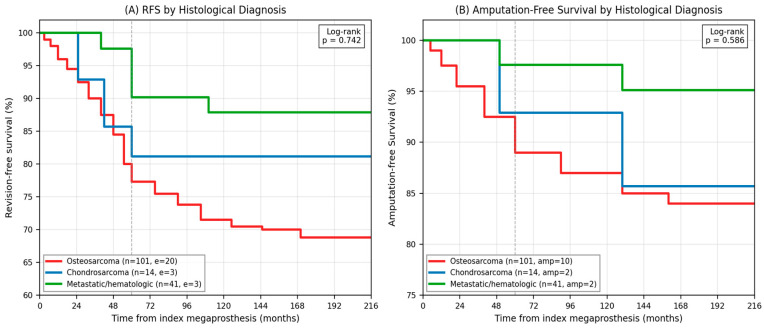
Exploratory Kaplan–Meier subgroup analyses by histological diagnosis (n = 156 across three groups). (**A**) RFS: osteosarcoma (n = 101, events = 20; 5-year RFS 77.3%), chondrosarcoma (n = 14, events = 3; 5-year RFS 81.2%), and metastatic/hematologic lesions (n = 41, events = 3; 5-year RFS 90.2%). (**B**) Amputation-free survival by diagnosis group. Log-rank *p* = 0.742 (RFS) and *p* = 0.586 (amputation-free). No statistically significant difference was identified across groups.

Exploratory Kaplan–Meier analyses were performed for the three largest histological subgroups with sufficient event counts for analysis: osteosarcoma (n = 101, 20 events), chondrosarcoma (n = 14, three events), and metastatic/hematologic lesions (n = 41, three events). The remaining histological categories (Ewing sarcoma, giant cell tumor of bone, other malignant sarcomas, benign/low-grade aggressive lesions, and not-otherwise-specified) were not analyzed separately because event counts were too small (≤2 per group) to support stable Kaplan–Meier estimation. Metastatic and hematologic lesions were grouped into a single analytic category due to small individual entity counts and shared selection criteria (adequate systemic disease control and expected survival exceeding 12 months). Five-year RFS was 77.3% (95% CI: 67.1–84.7%) for osteosarcoma, 81.2% (95% CI: 52.5–93.5%) for chondrosarcoma, and 90.2% (95% CI: 72.6–96.7%) for the metastatic/hematologic group. Log-rank testing showed no statistically significant difference across the three groups (*p* = 0.742). Similarly, amputation-free survival did not differ significantly by diagnosis (*p* = 0.586). Results are illustrated in Figure 4.

## 4. Discussion

This single-center series of 241 consecutive knee megaprostheses over 21 years provides systematic Kaplan–Meier survival data stratified by anatomical site and surgical margin, alongside the complete spectrum of unplanned revision surgeries. The principal findings are: (1) osteosarcoma accounted for 41.9% of a heterogeneous case mix that also included locally aggressive benign lesions, metastatic and hematologic lesions, and other sarcomas; (2) R0 margin was achieved in 85.5% of patients (206/241); (3) unplanned revision surgery affected 10.4% of patients, with meaningful limb salvage after debridement (77%) and recurrence resection (60%); (4) 5-year RFS of 73.8% (distal femur) and 65.0% (proximal tibia) and 10-year limb salvage of 88.9% and 79.2% are within ranges reported in published series, although direct cross-study comparisons should be interpreted cautiously given differences in case mix, follow-up, era, and failure definitions; and (5) within this cohort, surgical margin was the strongest independent predictor of both revision-free and amputation-free survival on multivariate analysis.

### 4.1. Distal Femur-Specific Comparisons

The distal femur group (*n* = 160) achieved a 5-year RFS of 73.8% and a 5-year limb salvage of 88.9%. The largest published single-site series, Pala et al. [11] from the Rizzoli Institute (*n* = 687, 1983–2010), reported implant survival of 70% at ten years, which is broadly in the same range as our 10-year RFS of 68.8%, although the two endpoints are not directly comparable (all failure types vs. first revision). The overall failure hierarchy in our series—infection-related debridement (Type 4, 11 patients, 6.9%) followed by aseptic loosening (Type 2, 8.1%)—is similar to that of the Rizzoli series. The R0 margin rate of 85.6% in the distal femur group is consistent with the standard of oncological wide resection at this site, and the margin effect observed on multivariate analysis (HR 2.14, *p* = 0.002) is broadly consistent with previously published estimates of approximately 1.8–2.5 [17]. The mean MSTS score of 23.8/30 (79.3%) in the distal femur group is in the range of values reported in comparable oncologic megaprosthesis series [11,17].

### 4.2. Proximal Tibia-Specific Comparisons

The proximal tibia group (n = 78) had a 5-year RFS of 65.0% (95% CI: 50.5–76.2%) and a 5-year limb salvage of 84.3% (95% CI: 70.8–91.9%), with non-significant trends toward inferiority versus the distal femur (*p* = 0.081–0.084). The 5-year RFS of 65.0% falls within the published range for proximal tibia series and is broadly consistent with the outcomes reported by Sacchetti et al. [10] once differences in follow-up duration are taken into account. Myers et al. [9] reported an approximately 10% amputation rate in 40 proximal tibia reconstructions at a mean of 95 months; this is in the same range as our 5-year amputation rate of 15.7% over a longer follow-up period. The absence of gastrocnemius flap coverage emerged as an independent predictor of failure in our multivariate analysis (HR 2.31, 95% CI 1.18–4.52, *p* = 0.015; Fine–Gray SHR 2.18, 95% CI 1.10–4.32, *p* = 0.025). Clinically, this association is biologically plausible: a rotational gastrocnemius flap obliterates the dead space anterior to the megaprosthesis, improves the soft tissue envelope over a region with intrinsically thin native coverage, and reduces the risk of wound dehiscence and secondary deep infection—the dominant failure mode at this site in our series and others [9,10,12]. These observations are consistent with Mavrogenis et al.’s [12] series, in which flap coverage emerged as an impactful technical factor for proximal tibia infection prevention. However, several considerations argue strongly for cautious interpretation of our hazard ratio. First, only 11 of the 78 proximal tibia patients (14.1%) underwent gastrocnemius flap coverage in our cohort, yielding limited statistical power to estimate this effect precisely; the wide 95% confidence interval (1.18–4.52) reflects this imprecision. Second, indication for flap coverage was not randomized: flaps were used selectively in cases the operating surgeon judged to be at higher risk for soft tissue compromise, introducing potential confounding by indication that a multivariate model with the variables we measured cannot fully eliminate. Third, the small flap subgroup (n = 11) precludes meaningful subgroup analysis by histology, era, or institutional learning curve. Our finding should therefore be regarded as supporting—rather than independently confirming—the existing literature on flap coverage; the hazard ratio we report should be interpreted as a directional signal whose true magnitude is best inferred from the broader literature, to which our data add a single-center observation rather than a definitive estimate. A larger multicenter cohort or systematic review with patient-level data would be required to provide a precise, generalizable effect estimate. The lower mean MSTS score in the proximal tibia group (21.4 ± 5.1 vs. 23.8 ± 4.2 for the distal femur; *p* = 0.018) reflects the functional impact of extensor mechanism disruption and is consistent with published proximal tibia series, in which MSTS scores typically range from 19 to 23 [9,10].

### 4.3. Combined-Site Comparisons and the Central Role of Surgical Margin

Against combined DF+PT series, our 7.1% amputation rate is at the lower end of published values: Bus et al. [13] 11%, Sevelda et al. [15] 21%, and Pala et al. [16] 29.1% overall failure at a mean four years; these comparisons should however be interpreted in light of differences in case mix, era, and definitions of failure. In a multicenter French series of 161 hinged knee reconstructions, Mazaleyrat et al. [14] reported a 5-year implant survival of 84% for the distal femur and 74% for the proximal tibia, with revision more frequently required in the proximal tibia (OR 2.4; 95% CI 1.2–5.1, *p* = 0.02), consistent with the site-specific risk pattern observed in our cohort. Within our cohort, surgical margin emerged as the strongest single covariate associated with revision-free and amputation-free survival, with a 5-year RFS gap of 75.4% (R0) vs. 48.7% (R1/R2; *p* < 0.001) and an amputation-free survival gap of 90.6% vs. 71.5% (*p* = 0.003). The hazard ratio of 2.14 for positive margin in multivariate Cox regression is in line with previously published estimates of approximately 1.8–2.5 [3,17]. Our findings are therefore best interpreted as confirming, in a single-center cohort outside the predominant Western series, the well-established association between surgical margin and outcome in bone sarcoma surgery, rather than as identifying a novel determinant. The clinical implication is that achieving a negative margin at index surgery should remain a high-priority technical goal in megaprosthetic reconstruction around the knee, while recognizing that intrinsic tumor biology and resectability—rather than margin alone—often constrain the achievable margin in any given case.

### 4.4. Revision Surgery in Context

The 10.4% revision surgery rate (36 procedures, 25 patients) is in the range reported in published sarcoma megaprosthesis benchmarks. In a comparative analysis of megaprosthetic reconstruction for primary sarcoma versus metastatic bone disease, Thorkildsen et al. [18] reported Kaplan–Meier revision rates of 18% at 1 year and 24% at 2 years in the sarcoma cohort—somewhat higher early rates consistent with the more biologically aggressive primary tumor population, although these revision-rate definitions differ from our composite revision-or-amputation endpoint. We chose a deliberately broad composite RFS endpoint (any unplanned revision or amputation) to capture the total secondary surgical burden experienced by patients; however, because the component events differ substantially in clinical severity, we performed a sensitivity analysis using a stricter major-event composite (amputation, implant removal, or recurrence resection only). The pattern of the primary findings—worse outcomes in the proximal tibia and with positive margins—was preserved under this stricter definition, suggesting that the primary RFS results are not driven primarily by minor revisions of limited severity. The 77% limb salvage rate after debridement and 60% after recurrence resection represent clinically important intermediate outcomes that are not uniformly reported in the existing literature [19].

**Limitations:** This study carries several limitations that should be borne in mind when interpreting the findings. First, as a retrospective single-center series from a tertiary national referral center, the cohort represents a selected population, and outcomes may differ from those achievable in community practice settings; the selection criteria for offering megaprosthetic reconstruction (Section 2.2) are explicitly tertiary-center criteria. Second, the cohort is histologically heterogeneous, encompassing high-grade malignant sarcomas, low-grade and benign aggressive lesions, and selected metastatic and hematologic disease within a single survival analysis; this heterogeneity reflects real-world practice but limits the biological interpretability of pooled survival estimates. We have presented exploratory subgroup analyses by histology (Section 3.7), but these are underpowered and hypothesis-generating only. Third, the composite RFS endpoint includes events of markedly different clinical severity; we have addressed this through a pre-specified major-event sensitivity analysis (Section 3.6), but composite endpoint heterogeneity remains an inherent limitation of any aggregate failure analysis. Fourth, MSTS functional scores were extracted from clinic notes rather than collected by a standardized instrument administered by an independent assessor, which introduces potential measurement bias. Fifth, Henderson classification data required retrospective extraction from operative and follow-up records, which may introduce documentation bias. Sixth, patients who underwent secondary procedures at external institutions may not have been fully captured in the institutional registry, potentially underestimating the true revision rate. Finally, certain subgroups underlying multivariate findings—most notably the gastrocnemius flap subgroup (n = 11)—are small, and the corresponding hazard ratios should be regarded as exploratory rather than as definitive estimates of independent effect.

## 5. Conclusions

In this single-center retrospective series of 241 consecutive knee megaprostheses over 21 years, megaprosthetic reconstruction around the knee was associated with acceptable long-term revision-free survival (5-year RFS 73.8% distal femur, 65.0% proximal tibia), limb salvage (5-year 88.9% and 84.3%, respectively), and functional outcomes (mean MSTS 23.8/30 distal femur, 21.4/30 proximal tibia). Positive surgical margin and proximal tibia location were associated with worse revision-free survival; within the proximal tibia subgroup, lack of gastrocnemius flap coverage was also associated with worse outcome on exploratory analysis. These findings reinforce the well-established importance of oncologic margin quality and site-specific soft tissue strategies in megaprosthetic reconstruction around the knee. Standardized multicenter analyses in histologically stratified cohorts, with prospectively collected functional outcomes and uniform failure definitions, are needed to refine the relative contributions of surgeon-modifiable and tumor-intrinsic factors to long-term outcome.

## Figures and Tables

**Table 1:** Demographic and follow-up characteristics by anatomical site (n = 241).

Variable	Distal Femur (n = 160)	Proximal Tibia (n = 78)	Combined * (n = 3)
Mean age ± SD (years)	36.7 ± 20.5	30.6 ± 16.8	51.0 ± 17.3
Median age, range (years)	29 (2–79)	24 (5–76)	42 (40–71)
Male/Female	85/75	51/27	3/0
Male, %	53.1%	65.4%	100%
Right/Left	74/86	40/38	2/1
Mean follow-up ± SD (months)	116.6 ± 65.2	130.1 ± 70.6	84.3 ± 42.7
Median follow-up, range (months)	95.6 (18.3–278.2)	110.0 (30.5–278.8)	76.4 (48.2–128.6)
Primary/Revision surgery	151/9	77/1	3/0

* Combined: 3 patients with simultaneous distal femur and proximal tibia reconstruction. These combined-site patients were excluded from site-stratified Kaplan–Meier analyses and from the whole-cohort Cox model (analytic cohort for these analyses n = 238), but were retained in cohort-wide descriptive summaries and in the unplanned revision and amputation summaries.

**Table 7:** Kaplan–Meier survival estimates by anatomical site and surgical margin.

Group	n	Ev.	1-Yr	2-Yr	5-Yr (95% CI)	10-Yr	*p* (Log-Rank)
RFS by Anatomical Site							
Distal Femur	160	22	88.0%	82.2%	73.8% (64.3–81.2%)	68.8%	0.084 *
Proximal Tibia	78	13	83.3%	74.4%	65.0% (50.5–76.2%)	58.1%	
Limb Salvage by Anatomical Site							
Distal Femur	160	10	93.7%	91.7%	88.9% (82.7–92.9%)	88.9%	0.081 *
Proximal Tibia	78	7	89.7%	87.2%	84.3% (70.8–91.9%)	79.2%	
RFS by Surgical Margin							
R0—Negative margin	203	22	89.3%	83.6%	75.4% (67.1–81.8%)	70.7%	<0.001
R1/R2—Positive margin	35	13	72.5%	59.9%	48.7% (27.9–66.6%)	39.0%	
Limb Salvage by Surgical Margin							
R0—Negative margin	203	11	94.4%	92.8%	90.6% (84.4–94.4%)	88.5%	0.003
R1/R2—Positive margin	35	6	82.5%	77.4%	71.5% (54.4–83.2%)	71.5%	

RFS: revision-free survival. * Indicates *p*-values that did not reach statistical significance (*p* ≥ 0.05) but suggested a clinical trend favoring the distal femur group. All confidence intervals computed using the Greenwood formula with log-log transformation.

## Data Availability

The data presented in this study are available on request from the corresponding author. The data are not publicly available due to patient privacy restrictions.

## Abbreviations

The following abbreviations are used in this manuscript:

RFS	Revision-free survival
DF	Distal femur
PT	Proximal tibia
MSTS	Musculoskeletal Tumor Society
HR	Hazard ratio
CI	Confidence interval
AKA	Above-knee amputation
